# Vestibular Cochlear Manifestations in COVID-19 Cases

**DOI:** 10.3389/fneur.2022.850337

**Published:** 2022-03-18

**Authors:** Kathiravan Kaliyappan, Yu-Chen Chen, Vijaya Prakash Krishnan Muthaiah

**Affiliations:** ^1^Department of Rehabilitation Sciences, School of Public Health and Health Professions, State University of New York at Buffalo, Buffalo, NY, United States; ^2^Department of Radiology, Nanjing First Hospital, Nanjing Medical University, Nanjing, China

**Keywords:** auditory symptoms, vestibular symptoms, COVID-19, hearing loss, tinnitus

## Abstract

The severe acute respiratory syndrome coronavirus 2 (SARS-CoV-2) is a high transmissible infectious disease that primarily impacts the respiratory system and leads to death as it worsens. Ever since the World Health Organization declared the disease as a global pandemic, the pathophysiology, clinical manifestations, and disease prognosis has been discussed in various literature. In addition to impaired respiratory health, the symptoms also indicated the involvement of the cardiovascular and neurological system after SARS-CoV-2 infection. Despite the pulmonary, cardiovascular, and neurological complications, many reports also revealed the prevalence of vestibulocochlear symptoms like dizziness, vertigo, vestibular neuritis, sudden sensorineural hearing loss, and tinnitus. Though many clinical reports and scientific reviews reported the vestibular and cochlear impairments associated with coronavirus disease 2019 (COVID-19) infection, the underlying pathological mechanisms are still unclear and unexplored. In this review, we discussed the published clinical reports, research articles, and literature reviews related to vestibulocochlear manifestations following SARS-CoV-2 infections. We also summarized the current knowledge about the prevalence, epidemiological and clinical features, and potential pathological mechanisms related to vestibular and cochlear manifestations resulting from COVID-19 infections.

## Introduction

The novel coronavirus disease 2019 (COVID-19) infection caused by severe acute respiratory syndrome coronavirus 2 (SARS-CoV-2), severely impairs the human respiratory system and its clinical manifestation includes cough, fever, and fatigues (1). In December 2019, the outbreak was identified as pneumonia cases with an unknown cause in Wuhan, China, the WHO declared the infection as a pandemic in March 2020 (2). As of March 2022, the pandemic has caused 440 million confirmed cases with 5.9 million deaths worldwide and is still persistent (https://covid19.who.int/).

Many countries are developing vaccines that mainly target the SARS-CoV-2 spike protein (S protein), a viral surface protein that enters the human cells (3). Even with significant protective measures, the disease is spreading relentlessly worldwide and causing health and socioeconomic burden. The COVID-19 pandemic also impacts the mental health of a larger population due to lockdowns and lifestyle modification-mediated stress, anxiety, depression, and insomnia (4).

The majority of COVID-19 infected cases are asymptomatic in the early stage of disease progression and affected predominantly with respiratory or extrapulmonary symptoms in subsequent stages (5). It is becoming apparent through clinical observations that patients with COVID-19 infection also exhibit neurological and otological symptoms (6, 7). With a high prevalent rate along with its asymptomatic and atypical nature, the COVID-19 is posing a huge burden and challenge to the healthcare communities. So there is a critical need for a detailed understanding of its demographics, transmission risk, symptoms, clinical outcomes, and manifestations in a broader manner.

In this review, we summarize the research reports and findings related to neurological, vestibular, and auditory complications in the patients affected with COVID-19 and discuss the potential pathological mechanisms.

## Review Methods

Case reports, case series, and multicentral studies were sought from PubMed and Google using keywords, “coronavirus or COVID and neurological symptoms, audiological symptoms including hearing loss, tinnitus, vestibular symptoms including dizziness and vertigo” were included for this review. Self-reported case reports and observational studies without any clinical examination or tests were excluded from this review.

## Neurological Manifestations in COVID-19 Patients

While COVID-19 infections primarily affect the respiratory system, many clinical observations and symptoms demonstrate its extrapulmonary involvement in cardiovascular, digestive, hematological, endocrine, excretory, and neurological systems (5, 8, 9). Recent evidence are indicating the increasing neurological manifestations in central and peripheral nervous systems with symptoms, including headaches, dizziness, fatigue, and loss of consciousness (10). In addition, other neurological manifestations like meningitis (11), encephalitis (12), encephalopathy (13), myelitis (14), and Guillain Barre syndrome (15), in patients with COVID-19, suggesting its neuroinfectious properties. Many clinical cases demonstrated the potential involvement of COVID-19 in acute ischemic stroke (AIS) (16–19). It is predicted that the stroke risk is increased to 3.2- to 7.8-fold after the first three days of COVID-19 infection (20, 21). Evidence also implied that COVID-19 infection may lead to arterial thrombosis through endothelial dysfunction, thrombin formation, and platelet activation (22). The neurological symptoms are identified as an initial COVID-19 presentation in many patients and the prevalence increases with the severity of the disease. The neurological manifestations and symptoms associated with COVID-19 are listed in Table 1.

**Table 1:** Neurological manifestations and symptoms in patients with COVID-19.

**Authors**	**Manifestations and symptoms**
Wang et al. (6)	Headaches, Dizziness, Fatigue
Bénézit et al. (23)	Hyposmia/anosmia and Hypogeusia/ageusia
Niazkar et al. (10)	Loss of consciousness
Liang et al. (11)	Meningitis
Ye et al. (12)	Encephalitis
Zhao et al. (14)	Myelitis
Filatov et al. (13)	Encephalopathy
Nannoni et al. (19)	Cerebrovascular disease
Sedaghat and Karimi (15)	Guillain Barre syndrome

## Otological Manifestations in COVID-19 Patients

The COVID-19 infection also presented with many early otolaryngological symptoms, like throat infections, dyspnoea, cough, along the sudden onset of anosmia and ageusia (23, 24). In addition, few case reports have documented the adverse otologic (25–28) and vestibular manifestations (29–31) after COVID-19 infection. The detailed otological information in each report was shown in Table 2.

**Table 2:** The detailed otological information in each report.

**Authors**	**Study design**	**Total patients**	**Age (Years)**	**Sex**	**Test**	**Symptoms**
Dharmarajan et al. (32)	Case series	100	21–60	Both	PTA & TEOAE	Tinnitus, Hearing loss & Otalgia
Sriwijitalai and Wiwanitkit (26)	Case report	1	NA	NA	NA	Neurosensory hearing loss
Abdel Rhman and Abdel Wahid (33)	Case report	1	52	Male	Audiometry	SNHL
Degen et al. (34)	Case report	1	60	Male	MRI	Acute SNHL
Edwards et al. (35)	Case report	1	68	Female	Audiogram	Bilateral SNHL
Koumpa et al. (36)	Case report	1	45	Male	PTA	Hearing loss
Fidan (37)	Case report	1	35	Female	Tympanometry & audiometry	Conductive hearing loss
Mustafa (25)	Case series	20	20-50	NA	Tympanometry & TEOAE	Mild hearing loss
Chirakkal et al. (38)	Case report	1	35	Female	PTA, Tuning fork test, Impedance audiometry, OAE & Tinnitus pitch matching	Tinnitus
Kokten et al. (39)	Case series	30	NA	NA	PTA & TEOAE	Hearing loss
Lamounier et al. (40)	Case report	1	67	Female	Audiometry & MRI	Hearing loss
Pokharel et al. (41)	Case report	1	27	Male	Audiometry & MRI	SNHL
Ricciardiello et al. (42)	Case series	5	18–65	Male & female	PTA, acoustic immittance test, ABR, Acufenometry, THI & DHI	SNHL & Tinnitus
Daher et al. (43)	Case report	1	49	Male	Tympanometry & audiometry	Severe tinnitus & Mild Hearing loss
Javanbakht and Babaee (44)	Case report	1	27	Male	Tympanometry & audiometry	Tinnitus
Sun et al. (45)	Case report	1	38	Male	Tympanometry	Bilateral hearing loss & tinnitus
Viola et al. (46)	Multicentral study	185	NA	NA	Questionnaire	Tinnitus, Vestibular disorders, Dizziness & Vertigo
Liotta et al. (47)	Case series	509	NA	NA	Clinical examination	Neurologic manifestations & encephalopathy
Sari et al. (48)	Case report	2	12–13	Boy & Girl	NA	Headache & Dizziness
Vanaparthy et al. (49)	Case report	1	63	Female	Dix-Hallpike maneuver	Vertigo and vestibular neuritis
Maslovara and Košec (50)	Case report	2	29 & 41	Female	The Romberg test & Dix–Hallpike test	Vertigo, dizziness, and nausea
Mat et al. (51)	Case report	1	13	Female	Vestibulo-ocular reflex	Vestibular Neuritis
Motawea and Monib (52)	Case report	1	60	Female	Physical examination	Vertigo
Malayala and Raza (53)	Case report	1	29	Female	Self-report & Clinical examination	Vestibular Neuritis

*PTA, pure tone audiometry; TEOAE, transient evoked otoacoustic emissions; ABR, auditory brainstem response; THI, tinnitus handicap inventory; DHI, dizziness handicap inventory; VOR, vestibulo-ocular reflex; NA, not applicable*.

## Auditory Manifestations

Hearing loss after viral infections are conductive or sensorineural types caused and being hypothesized to cause direct or indirect damage to inner ear structures. Studies have highlighted the neurotropic and neuroinvasive nature of the COVID-19 virus (54), and the infection has been considered a plausible cause for hearing loss. Auditory symptoms, including hearing loss and tinnitus, are being frequently reported along with other usual symptoms in patients with COVID-19 (32, 55, 56). Being a neglected symptom, screening for hearing is being encouraged in the patients with COVID-19. The first case of sensorineural hearing loss (SNHL), in a COVID-19-recovered elderly female patient, was reported in Thailand (26). Following this, a few other reports also stated the association between the SNHL and COVID-19 infection (33–36). Similarly, a case report demonstrated a unilateral conductive hearing loss and tinnitus in a 35-year-old female asymptomatic patient with COVID-19. The otoscopic examination revealed the acute otitis media mediated bulging tympanic membrane (37). In another study, asymptomatic patients with COVID-19 have significantly worsened high-frequency pure tone thresholds and transient evoked otoacoustic emission (TEOAE) amplitudes when compared to control subjects (25). In addition, a recent also study confirmed hearing impairment especially at 1,000 Hz through TEOAE tests (39).

Tinnitus is another significant clinical symptom additional to hearing loss, vertigo, and dizziness in patients with COVID-19 infection. Chirakkal et al. (38) have reported that unilateral tinnitus was observed at 4 kHz at 10 dB using frequency and intensity matching evaluation in a 35-year-old female patient with COVID-19. This study also demonstrated the detrimental effects of COVID-19 infection on cochlea outer hair cells, which was evident through TEOAE and distortion-product otoacoustic emissions amplitudes. Many case reports and reviews have reported sudden hearing loss and disabling tinnitus in a patient with severe COVID-19 infection (27, 40–42, 57). Furthermore, sudden unilateral hearing loss with worsening tinnitus was observed in a 52-year-old male physician affected with COVID-19 without any history of head trauma or ototoxic medications (33). Another study has reported bilateral tinnitus in a 60-year-old patient with COVID-19 having signs of inflammation in both cochleae through MRI findings (34). Multiple case reports and meta-analysis reviews have shown the prevalence of tinnitus in patients with COVID-19 (43–46, 56, 58, 59).

A case of sensorineural hearing loss and tinnitus was reported in the unilateral ear 2 days after administration of the Oxford-AstraZeneca (VAXZEVRIA) vaccine in a 57-year-old male patient (60). In another case study, the SSHL was demonstrated through pure tone audiometry 2 days after the second dose of Oxford-AstraZeneca vaccine in a 61-year-old female (61). But the hearing loss was recovered after 15 days with a treatment of glucocorticoids and acetylsalicylic acid. Furthermore, a 37-year-old male patient was diagnosed with tinnitus and cochleopathy after receiving his first AstraZeneca COVID-19 vaccine dose, which was reversed by dexamethasone and prednisolone treatment (62). Another case series have reported the transient sudden unilateral tinnitus after BNT162b2 mRNA-vaccine (e.g., *pfizer*), which resolved rapidly in 2 out of 3 cases (63). Recently, many case reports have reported a prevalence of sudden hearing loss and tinnitus after the COVID-19 vaccinations (64–68).

## Vestibular Manifestations

Though the involvement of COVID-19 infection in otologic manifestations has not been confirmed yet, many case reports are providing preliminary evidence to emphasize the potential association between the COVID-19 infection and ear disorders. Along with auditory manifestations, a few vestibular symptoms like dizziness, vertigo, and tinnitus are described as the common clinical manifestations in patients with COVID-19 (47, 69). Many case studies have reported dizziness as a prevalent neurological symptom post-COVID-19 infection (47, 69, 70). In addition, many other reviews also reported dizziness as a common clinical manifestation along with other vertigo, hearing loss, and tinnitus (56, 71). A recent case report has stated the manifestation of dizziness in two COVID-19-affected children (12–13 years), which resolved in a week (48). Vestibular neuritis is a vestibular disorder that causes vertigo, dizziness, and balance problems, and is diagnosed in patients with COVID-19 (49). In addition, a few case reports also demonstrated vertigo as an important clinical manifestation of COVID-19 (50–53).

## Discussion

Association between various types of viral infections and hearing loss have been implied for years. Hearing loss is a well-known complication of bacterial and viral meningitis (72) and some viral infections can cause SNHL (73, 74).

Many researchers have discussed the neuroinvasive and neurotropic properties of SARS-CoV-2 (54, 75, 76), which has been linked the post-COVID neurological manifestations. Almost every coronavirus variant has a similar structure and infection mechanisms. Earlier reports have confirmed the presence of SARS-CoV in cerebrospinal fluid of infected patients (77, 78).

SARS-CoV-2 primarily enters the body through the angiotensin-converting enzyme-2 receptor (ACE2) in the respiratory epithelium. The virus is replicated and enters the circulation by attaching to the β chain of hemoglobin in erythrocytes, and transported and binds to several organs (79).

**Figure 1:**
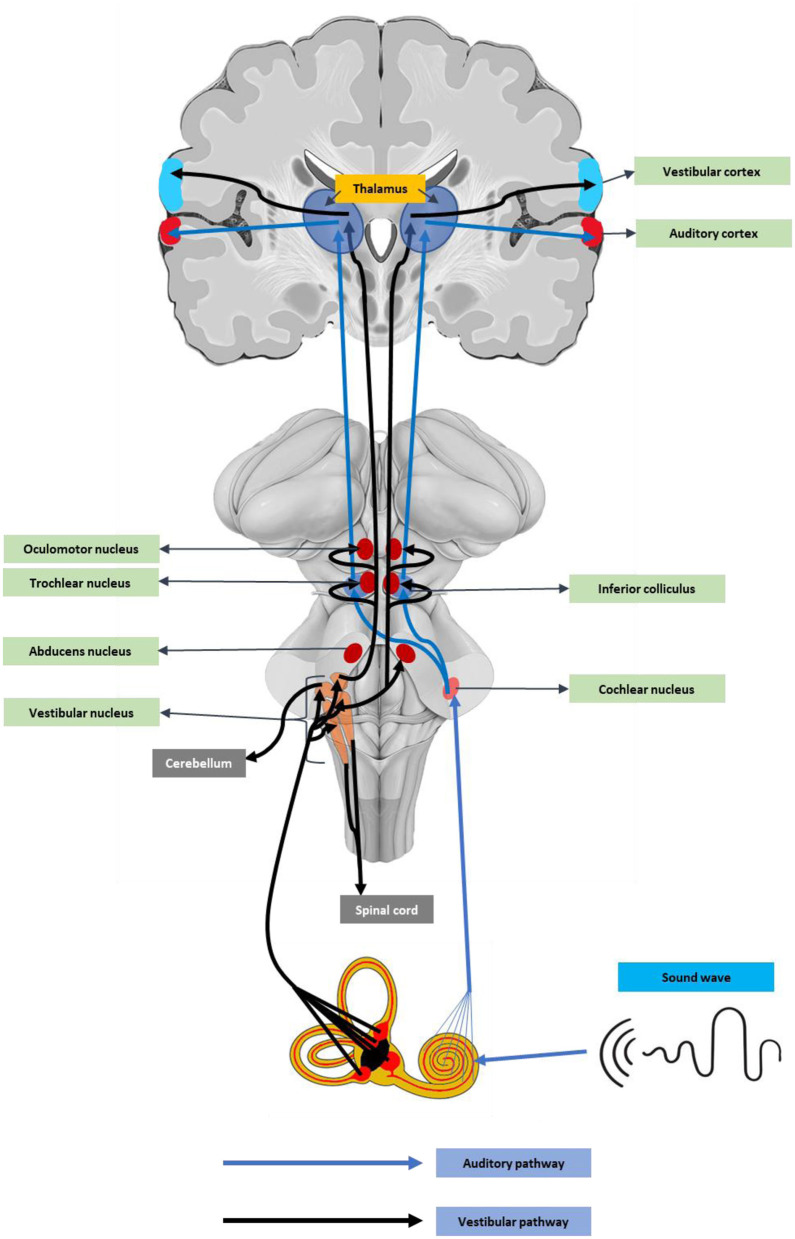
Vestibulocochlear pathway.

Angiotensin-converting enzyme-2 receptor (ACE2) receptors are abundant in neurons and glial cells of various brain regions like the cortex, striatum, substantia nigra, and the brainstem (80), suggesting the neuronal damage potential of SARS-CoV-2. In addition, the ACE2 receptors, including the medulla oblongata and temporal lobe (81), are key auditory regions. These auditory centers could be affected by cytokine release-mediated inflammatory responses (82). Recent reports also stated that SARS-CoV-2 can directly enter the brain through the olfactory epithelium or the cribriform bone (83). An earlier experimental study using a human ACE2 overexpressed mouse model, the intranasal SARS, SARS-CoV-1 infection caused neuronal death in the brainstem regions (84, 85). As the brainstem contains vital components of the auditory pathway (Figure 1), damage to these regions can cause hearing complications and deafness.

The blood-brain barrier is a physical barrier that prevents the entry of any harmful and infectious substances. Recent evidence also demonstrated that the spike protein of SARS-CoV-2 was able to cross the blood-brain barrier in male mice when it is administrated intravenously and intranasally (86). Adding to this, many research reports and review articles also cite the evidence of blood-brain barrier disruption caused by SARS-CoV-2 infection through upregulation of proinflammatory mediators (87–90). With a compromised blood-brain barrier, the SARs-CoV-2 can easily enter the brain parenchyma and lead to exacerbated brain pathology and neurological manifestations, including hearing and vestibular complications.

Another possible hypothesis of hearing loss and vestibular complications is hypoxia caused by the hyperfusion-mediated ischemia in the inner ear structures. Numerous clinical and experimental reports have confirmed that the SARS-CoV-2 infection enhances the chances of thrombus formation in the circulation and leads to increased risk for cerebrovascular diseases (91–96). SARS-CoV-2 can cause hypoxia by deoxygenating the binding erythrocytes. As vascular smooth muscles contain ACE2 receptors, the SARS-CoV-2 infection can form a blood clot in the blood vessels supplying the inner ear, thus leading to ischemic damage and subsequent hearing loss and vestibular impairments. According to the ischemia theory, the geriatric population is mainly prone to SARS-CoV-2-mediated otological complications (97).

A recent research finding has demonstrated the involvement of direct viral infections in the inner ear tissues as a potential cause for auditory and vestibular dysfunctions after COVID-19 infection (98). Varying degree of hearing impairment by direct or indirect damage to inner ear components following viral infections can be reversed with antiviral drugs. But, ototoxicity by specific drugs to treat SARS-CoV-2 infection, could be a potential cause for negative auditory and vestibular manifestations of COVID-19 treatments. Drugs like hydroxychloroquine used in early pandemics were proved to be ototoxic and cause SNHL, tinnitus, and balance issues (99). In addition, other drugs like azithromycin (100), Remdesivir, Favipiravir (101), and Lopinavir (102) used to combat COVID-19 have been proved to cause ototoxicity.

## Conclusion

The novel coronavirus disease 2019 (COVID-19) pandemic became the major healthcare challenge in recent human history. Although respiratory and cardiovascular are identified as characteristic features, neurological and otological manifestations are being frequently reported in patients with COVID-19. These atypical symptoms can severely affect the long-term outcomes and impair the post-COVID-19 life quality. However, current data available on inner ear disorders associated with COVID-19 and studies describing the possible pathophysiology remain unclear and limited. Thus, there is a critical need for early screening and clinical laboratory diagnosis to identify the auditory and vestibular disorders to manage the disease effectively. Furthermore, it is crucial to ascertain the potential ototoxic properties of the drug used to manage COVID-19 to avoid permanent hearing and vestibular disorders.

## Author Contributions

KK checked the references and wrote the manuscript. Y-CC and VK contributed to the discussion and manuscript revision. All authors contributed to the article and approved the submitted version.

## Funding

This work was supported by the Natural Science Foundation of Jiangsu Province (No. BK20211008) and Medical Science and Technology Development Foundation of Nanjing Department of Health (No. ZKX20037).

## Conflict of Interest

The authors declare that the research was conducted in the absence of any commercial or financial relationships that could be construed as a potential conflict of interest.

## Publisher's Note

All claims expressed in this article are solely those of the authors and do not necessarily represent those of their affiliated organizations, or those of the publisher, the editors and the reviewers. Any product that may be evaluated in this article, or claim that may be made by its manufacturer, is not guaranteed or endorsed by the publisher.
